# Educational tools that address pregnancy‐related complications in the postpartum period: A scoping review

**DOI:** 10.1002/pmf2.70327

**Published:** 2026-08-03

**Authors:** Skylar N. Smith, Chloe A. Zera, Emma M. Anghel, Michelle C. Lara, Giselle Chiprez Ramirez, Carrie G. Wade, Ann C. Celi

**Affiliations:** ^1^ Department of Medicine Division of General Medicine and Primary Care Brigham and Women's Hospital Boston Massachusetts USA; ^2^ Department of Obstetrics and Gynecology Division of Maternal‐Fetal Medicine Beth Israel Deaconess Medical Center Boston Massachusetts USA; ^3^ Department of Obstetrics Gynecology, and Reproductive Biology Harvard Medical School Boston Massachusetts USA; ^4^ Countway Library Harvard Medical School Boston Massachusetts USA; ^5^ Department of Medicine Division of Women's Health Brigham and Women's Hospital Boston Massachusetts USA; ^6^ Department of Medicine Harvard Medical School Boston Massachusetts USA

**Keywords:** patient education, postpartum education, postpartum period, pregnancy complications

## Abstract

Previous research provides evidence that the postpartum period is a crucial time for individuals who have just given birth to understand and engage in their health to mitigate current and future risks. The present study is a scoping review of research on educational tools that address pregnancy‐related complications for postpartum patients. We aimed to determine the extent to which these tools exist and how they have been studied among postpartum people. PubMed, CINAHL (Cumulative Index to Nursing and Allied Health Literature), and Embase were searched from inception to June 2025 to identify publications on patient‐facing educational tools for the postpartum period that focus on pregnancy‐related complications. Educational tools were evaluated for application to the Capability, Opportunity, and Motivation (COM‐B) framework for health behavior change. Of 3216 records, 32 studies met the inclusion criteria, and 11 patient educational tools were accessible for analysis. Two reviewers independently screened and assessed the titles, abstracts, and full texts of identified articles for inclusion. These tools were delivered through a variety of media, including mobile applications and traditional written pamphlets. Topics of the tools included postpartum mental health, hypertensive disorders of pregnancy, gestational diabetes, breastfeeding after a complicated pregnancy, and warning signs for postpartum morbidity and mortality. Enhancers to postpartum care were addressed more often in the educational tools than barriers to postpartum care. The educational tools most often covered the following facilitators of behavior change identified by the COM‐B framework: psychological capability, social opportunity, and reflective motivation. This scoping review highlights the limited availability of educational tools for postpartum care and recovery that address pregnancy‐related complications. Findings of this scoping review suggest that educational tools that address pregnancy‐related complications for postpartum patients were limited in topics covered, language availability, and diversity of postpartum populations tested. Further research is needed to expand the network of accessible postpartum educational tools and to assess the efficacy and acceptability of these educational tools in historically marginalized populations. Structuring these tools within a framework for behavior change such as COM‐B may be a useful method for better understanding and improving postpartum care engagement and access to educational resources.

## 1 INTRODUCTION

### 1.1 Background/rationale

Maternal morbidity and mortality remain critical health issues in the United States and globally, particularly in the weeks and months after delivery. In 2023, approximately 700 birthing people died per day from preventable, pregnancy‐related causes [1]. This indicates a global maternal mortality ratio (MMR) of 197 maternal deaths per 100,000 livebirths [1]. While the overall global MMR has steadily declined (−40%) since 2000, more work is needed to meet the United Nations (UN) Sustainable Development Goal Target 3.1 by 2030, which aims for a global MMR of fewer than 70 deaths per 100,000 live births [1].

The United States (US) has the highest MMR among high‐income countries [2]. In the US, Black and Indigenous birthing people are disproportionately affected, with MMRs over three times higher than those of their white counterparts [3, 4]. Over 80% of pregnancy‐related deaths are preventable with the proper treatment of mental and physical disorders, and over half of pregnancy‐related deaths occur 7–365 days after delivery [5], suggesting the need for improved interventions in the postpartum period. Furthermore, birthing people who experienced severe morbidity during pregnancy are at an increased risk of mortality during the year following delivery [6].

The heightened mortality risk among birthing people with complicated pregnancies [7]—characterized by preexisting medical conditions, obstetrical complications (e.g., hypertensive disorders, preterm delivery), or fetal/neonatal complications (e.g., congenital anomalies, fetal growth abnormalities)—underscores the necessity of targeted physical and mental health interventions for the postpartum period [8]. The postpartum period provides an important opportunity to improve the care of birthing people who experienced—or are at‐risk for—adverse pregnancy outcomes and facilitate care transitions to comprehensive health and social services, including primary care, mental healthcare, and support for food, economic, housing, or other socioeconomic needs [9].

However, many birthing people, particularly those most at‐risk and who are often from underserved communities (e.g., from low socioeconomic backgrounds or who identify as Black, Indigenous, or people of color), do not receive adequate postpartum healthcare [10, 11]. Postpartum birthing people traditionally attend one visit with their obstetric provider around six weeks after delivery [9]. In reality, postpartum visit attendance is poor (60%) [8], and a single visit is often insufficient to comprehensively address the needs of postpartum people [9]. Furthermore, while primary care engagement is recommended in the year postpartum and is key to maintaining long‐term health beyond the postpartum period [8], postpartum people do not always experience a warm handoff and proper transition to primary care [9].

Optimizing postpartum care requires extending support beyond the clinic and into the hands and minds of postpartum people. Patient health education can be a vital tool for promoting positive health management behaviors (e.g., healthy eating, adequate sleep, engagement in postpartum care) and preventing adverse health outcomes in birthing people [12, 13, 14]. Despite the value that postpartum education can provide, birthing people often lack knowledge about the postpartum period (e.g., warning signs of urgent medical complications) after delivery due to more systemic issues, such as not receiving health education on a specific topic, language barriers, or cultural incompatibility of the health education [14, 15].

Effective postpartum health education aims to promote healthy behaviors in birthing people by targeting factors that affect their engagement in their postpartum care and recovery [16]. The Capability, Opportunity, Motivation, Behavior (COM‐B) model provides a framework for understanding the interaction between personal and environmental factors that shape the behavior of an individual [17]. According to the COM‐B framework, an individual must possess sufficient capability, motivation, and opportunity to demonstrate a specific behavior. COM‐B incorporates six components of behavioral change: physical capability (skills), psychological capability (knowledge), physical opportunity (environment), social opportunity (sociocultural norms), automatic motivation (emotions and impulses), and reflective motivation (evaluation and planning) [17]. The COM‐B framework has been utilized in healthcare settings, including obstetrics, to create successful health interventions [18], such as promoting healthy lifestyle changes to address gestational diabetes [19] and physical activity during pregnancy [20]. Using the COM‐B model to develop and analyze postpartum health education can improve its ability to target and address the barriers and facilitators that birthing people experience when engaging in postpartum care and recovery.

However, it is unclear what types of educational tools are available for the postpartum period that focus on pregnancy‐related complications and have been studied among birthing people for their acceptability, utility, and effect on postpartum health and behavioral outcomes. A comprehensive analysis of postpartum educational tools for pregnancy‐related complications in engendering and sustaining healthy behaviors is lacking in the literature.

### 1.2 Objectives

The primary aim of this paper was to complete a scoping review examining the availability of patient educational tools that seek to (1) support recovery and future health outcomes after a complicated pregnancy or (2) prevent further pregnancy‐related complications in the postpartum period. These complications included, but were not limited to, maternal mental health conditions, gestational diabetes, labor complications, and hypertensive disorders of pregnancy (HDPs). All educational tools included were evaluated for their potential for behavior change using the COM‐B framework. Given the heterogeneity of outcomes evaluated across studies, our review focused on the overall intent and mechanisms of behavior change rather than specific outcomes among birthing people. This review also sought to identify existing gaps in the research on postpartum educational tools and in the information provided in these educational tools for birthing people.

## 2 METHODS

A scoping review of the published literature on educational tools that address pregnancy‐related complications for postpartum people was conducted. This scoping review was reported in accordance with the Preferred Reporting Items for Systematic Reviews and Meta Analyses Extension for Scoping Reviews (PRISMA‐ScR) guidelines [21, 22]. This review was registered on the Open Science Framework (OSF) (Registration doi available at https://doi.org/10.17605/OSF.IO/CE7QK). The search was completed in June 2025.

### 2.1 Search strategy

We searched the following electronic bibliographic databases to identify relevant publications: PubMed, CINAHL (Cumulative Index to Nursing and Allied Health Literature), and Embase. In consultation with a medical librarian, the team drafted a search strategy using robust terminology for pregnancy and delivery complications, patient education, and the postpartum period. Appendix S1 provides the search strategy used in the review. Publications identified through the database search strategies were exported to the reference management application Zotero, and duplicate publications were removed.

### 2.2 Eligibility criteria

Publications must have focused on and studied the efficacy of patient‐facing educational tools that focus on pregnancy‐related complications in the postpartum period. These complications included, but were not limited to, maternal mental health conditions, gestational diabetes, HDPs, and labor complications. Publications were excluded if they did not present original research on a patient educational tool. Studies were also excluded if the educational tool was not provided or linked in the article itself, and if we were unable to access the tool when contacting the authors of the study.

*Population*. Present data on birthing people in the postpartum period.
*Concept*. Must describe at least one patient‐facing educational tool that addresses pregnancy‐related complications for postpartum patients. Any relevant tool topics may be included, such as postpartum mental health, hypertension, symptoms of other health complications, and resources for social and economic concerns. Must have collected and analyzed original data on patient outcomes related to the tool(s), such as health, behavioral, or knowledge‐based changes.
*Context*. Publications and patient educational tools from any country and publication year up to June 2025. Available in English, Spanish, or French due to language limitations of the research team.
*Types of evidence source*. Primary studies of any design.

### 2.3 Data screening

Two reviewers independently assessed and screened the titles, abstracts, and full texts of identified articles for inclusion and charted this using a spreadsheet developed by the research team. Articles were screened to ensure the inclusion of a patient‐facing educational tool; a tool that addresses pregnancy‐related complications; and an analysis of the educational tool being tested on postpartum patients. If all eligibility criteria were met, the independent reviewer listed the article as eligible to be included in the scoping review. A third reviewer resolved discrepancies through independent assessment and screening.

### 2.4 Data synthesis

The characteristics of studies that reported on an educational tool designed to address pregnancy‐related complications in the postpartum period were summarized. The articles were narratively synthesized. The type of pregnancy‐related complication addressed in each educational tool was identified, as well as the medium of the tool, the language of the tool, and the accessibility of the tool. The study design and methods of each study were also summarized.

### 2.5 Data analysis using the COM‐B framework

For included studies with readily accessible educational tools, a spreadsheet was developed to map and analyze the educational tools according to the COM‐B framework. This spreadsheet was based on a codebook with the principles of COM‐B previously used in research [23]. Two reviewers independently analyzed each educational tool according to the components of COM‐B. A third reviewer resolved discrepancies through independent assessment and screening.

## 3 RESULTS

**FIGURE 1:**
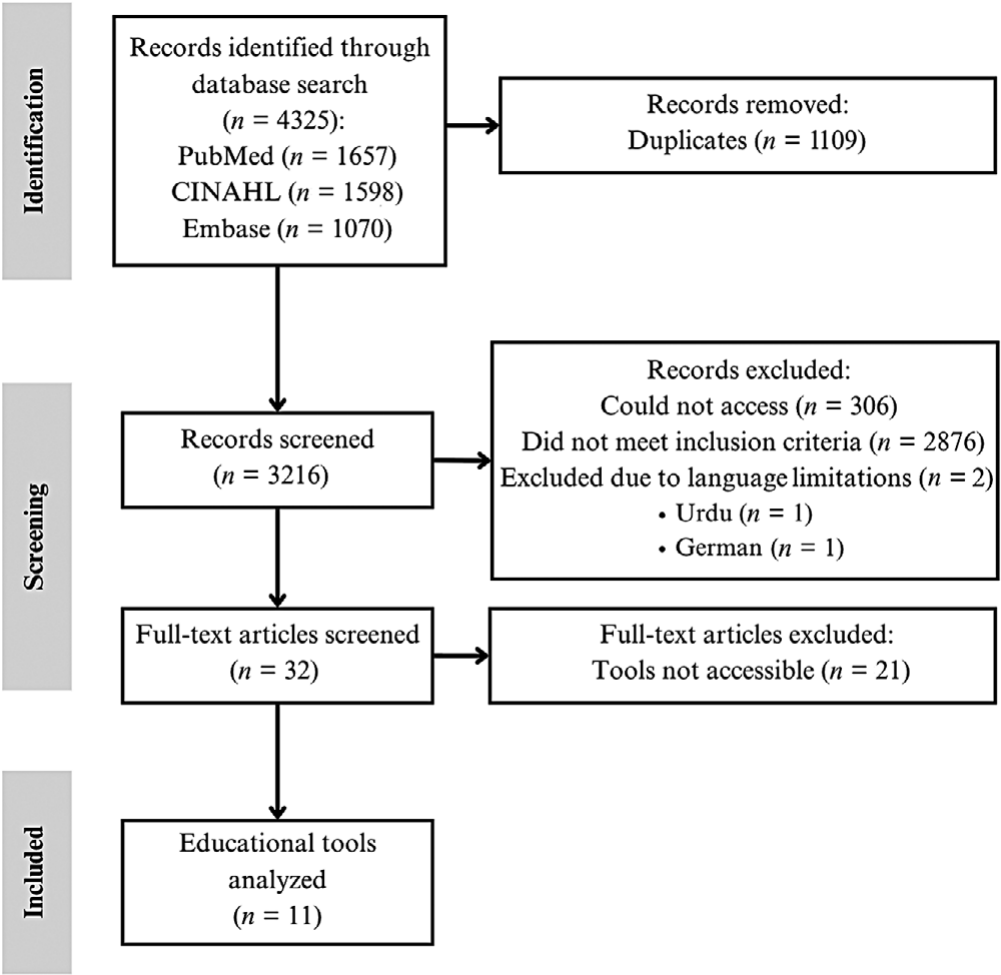
PRISMA flow diagram. CINAHL, Cumulative Index to Nursing and Allied Health Literature; PRISMA, Preferred Reporting Items for Systematic Reviews and Meta Analyses.

The search strategy identified 3216 publications for screening, of which 32 studies met the inclusion criteria, and 11 patient educational tools were accessible for analysis. Figure 1 shows the PRISMA flow diagram for this review. Table 1 summarizes the characteristics, studied outcomes, and COM‐B sub‐categories of all educational tools included in the analysis.

**TABLE 1:** Characteristics, outcomes, and COM‐B characteristics of all educational tools included.

Reference	Authors (date)	Topic	Subtopic	Outcomes measured	COM‐B enhancers addressed	COM‐B barriers addressed
[24]	Parlier‐Ahmad et al. (2023)	Mental health	Opioid use disorder	Implementation	Psychological capability, social opportunity	Psychological capability, reflective motivation, social opportunity
[25]	Fisher et al. (2016)	Mental health		Clinical	Physical capability, psychological capability, automatic motivation, reflective motivation, social opportunity	Psychological capability, reflective motivation
[26]	Mitchell et al. (2018)	Mental health	Self‐compassion	Implementation	Physical capability, psychological capability, automatic motivation, reflective motivation	Physical capability, reflective motivation
[27]	Parfenova et al. (2021)	Hypertensive disorders of pregnancy		Knowledge	Psychological capability, reflective motivation, social opportunity	
[28]	Sukumar et al. (2018)	Gestational diabetes mellitus		Behavioral & clinical	Psychological capability, automatic motivation, social opportunity	
[29]	Heh & Fu (2003)	Mental health	Postpartum depression	Clinical	Physical capability, psychological capability, reflective motivation, social opportunity	Automatic motivation, social opportunity
[30]	Daehn et al. (2023)	Mental health	Postpartum depression	Implementation	Physical capability, psychological capability, automatic motivation, social opportunity	
[31]	Timmer‐Hawck (1998)	Breastfeeding		Implementation	Psychological capability, automatic motivation	
[32]	De Los Reyes (2022)	Maternal morbidity and mortality	Severe maternal morbidity	Knowledge	Psychological capability, reflective motivation, social opportunity	
[33]	White et al. (2023)	Maternal morbidity and mortality	Maternal mortality	Knowledge	Psychological capability, social opportunity	
[34]	Stierman et al. (2024)	Maternal morbidity and mortality	Urgent maternal warning signs	Implementation	Psychological capability	

Abbreviation: COM‐B, Capability, Opportunity, Motivation, Behavior.

### 3.1 Educational tool characteristics

The most common forms of educational tools were videos, written materials (e.g., pamphlet, flyer), mobile applications, and online learning modules. Most of the educational tools were developed or available in English (*n* = 10).

Many of the educational tools focused on specific physical or mental health concerns or diagnoses. Postpartum mental health (*n = *5), including postpartum depression and opioid use disorder, was the most common topic discussed in the educational tools. The other most common topic addressed by the educational tools was postpartum maternal morbidity and mortality (*n = *3). Gestational diabetes, HDPs, and breastfeeding following a complicated pregnancy were each addressed by one tool, respectively.

### 3.2 Outcomes of testing the educational tools

The educational tools were studied in birthing people for a variety of outcomes, including implementation (e.g., perceived utility and acceptability of the tool), knowledge (e.g., knowledge retention after exposure to the tool), behavioral (e.g., physical movement changes after exposure to the tool), and clinical (e.g., changes in biomarkers and mental health scores after exposure).

### 3.3 Educational tools according to the COM‐B framework

**FIGURE 2:**
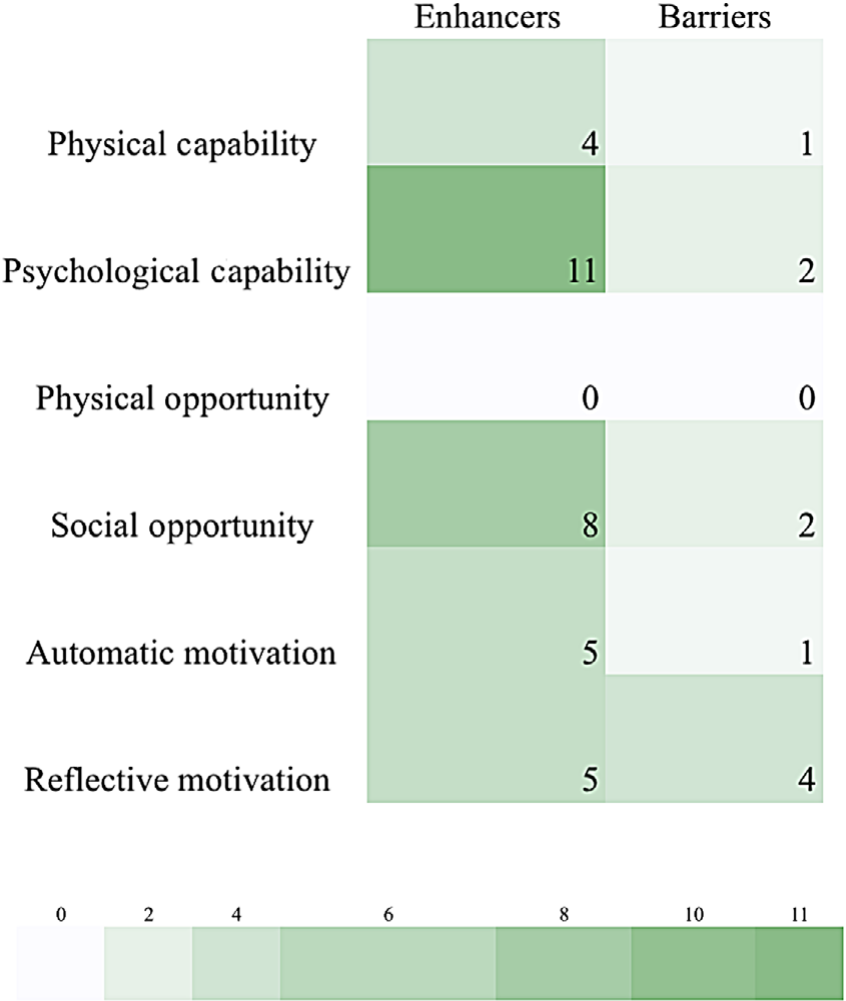
Heatmap demonstrating the frequency of educational tools categorized by capability, opportunity, and motivation sub‐categories.

Within the COM‐B framework, the educational tools addressed enhancers to postpartum care more often than discussing barriers to care. The facilitators of behavior change that were most frequently addressed in the educational tools were psychological capability (i.e., enhanced knowledge about postpartum care and recovery) and social opportunity (i.e., encouragement and social influences to improve postpartum care and recovery). Figure 2 provides a heatmap illustrating how often the educational tools addressed the respective capability, opportunity, and motivation sub‐categories.

### 3.4 Capability

Capability barriers to postpartum care were addressed in three educational tools. Psychological capability barriers to postpartum care included birthing people lacking the knowledge to properly address their postpartum needs, such as not understanding their baby's cry or how to access care in the postpartum period [24, 25]. Physical capability barriers to postpartum care addressed in the educational tools included birthing people not having the skills to address their mental health needs [26]. Psychological capability enhancers to postpartum care were focused on most often, with every tool promoting the enhancement of knowledge surrounding postpartum care [24, 25, 26, 27, 28, 29, 30, 31, 32, 33, 34]. Physical capability enhancers to postpartum care in the educational tools focused on skills postpartum people can use to improve their mental health and well‐being, such as meditation and self‐compassion exercises [26].

### 3.5 Opportunity

Opportunity barriers and enhancers to postpartum care were often addressed in the educational tools, with a focus on social opportunity enhancers. Social stigma was the most common social opportunity barrier to postpartum care addressed in the educational tools [24, 29]. Social opportunity enhancers to postpartum care were more common in the educational tools than physical opportunity enhancers, particularly the means to form and rely on social networks of personal loved ones or healthcare providers [24, 25, 27, 28, 29, 30, 32, 33]. Physical opportunity enhancers were not focused on in the educational tools.

### 3.6 Motivation

Motivational barriers to care were addressed less often in the educational tools than motivational enhancers. Reflective motivational barriers, such as negative beliefs and stigma about their capability to engage in postpartum care and recovery [24, 25, 26, 33], were focused on more often than automatic motivational barriers, which included feelings of hopelessness and guilt in birthing people related to help‐seeking [29]. Both reflective and automatic motivational enhancers to postpartum care were often addressed in the educational tools. Reflective motivational enhancers involved goal setting [27] and a content focus on encouragement and empowerment for engaging in postpartum care and recovery [25, 26, 29, 32]. The most predominant examples of an automatic motivational enhancer were mobile applications with motivational messaging for postpartum birthing people and self‐tests for birthing people to measure their progress throughout the educational programs [25, 28, 30, 31].

## 4 DISCUSSION

This scoping review identified the facilitators and barriers to postpartum care addressed in educational tools that focus on pregnancy‐related complications for postpartum patients within a structured framework for behavioral change. The educational tools most often enhanced the knowledge needed to access and participate in postpartum care and recovery (psychological capability) and encouraged the use of social support systems to assist with postpartum care access (social opportunity). Most categories and sub‐categories of the COM‐B framework were covered by the educational tools, suggesting a more widespread consideration of common barriers and facilitators to postpartum care and recovery among the educational tools. However, all sub‐categories of the COM‐B framework were not covered equally. For example, automatic motivational (emotions and impulses) barriers to postpartum care were addressed less often in the tools, despite the prevalence of mental health issues in the postpartum period [35, 36].

The topics covered by the educational tools were also limited in scope, focusing primarily on mental health conditions and warning signs for maternal morbidity and mortality. There was a distinct dearth of educational tools on postpartum care and recovery for patients who experienced adverse pregnancy outcomes, such as HDPs and gestational diabetes. HDPs and gestational diabetes are common causes of complicated pregnancies [37, 38, 39] and are linked to further complications during and beyond the postpartum period [40], indicating a need for educational tools on these topics for birthing people.

Furthermore, few of the studied educational tools explicitly focus on the social determinants of health (SDOH) that impact individuals in the postpartum period. Factors like maternal race, food and housing insecurity, as well as lack of insurance, transportation, and social/community support may all influence postpartum care and access [41, 42, 43] and are especially important to address when navigating pregnancy complications. While several of the educational tools identified in this scoping review address stigma and emphasize seeking social support, many SDOH are underrepresented. The American College of Obstetrics and Gynecology (ACOG) has emphasized the importance of assessing and addressing the SDOH of birthing people to improve postpartum care and its access [8, 44]. Therefore, educational tools designed to address pregnancy related complications in the postpartum period could be strengthened by providing resources on prescient SDOHs, such as insurance navigation and affordable food and housing recourses for new parents. Since all SDOHs affecting birthing individuals may be difficult to address in a patient educational tool, another way to ensure the equitable nature of these resources would be to test their acceptability and feasibility amongst underserved postpartum populations. Few articles in this scoping review explicitly tested their educational tools in diverse patient populations. Future research should focus on the development and testing of educational tools among a wider array of postpartum patient populations, especially those from marginalized backgrounds who are most likely to experience pregnancy complications [45, 46] and most likely to be impacted by SDOH which many be affecting their postpartum care.

The tools were developed using various media, from mobile applications to traditional written pamphlets. In the current healthcare environment [47], online and mobile educational tools are increasingly more important for birthing people to have constant access to the knowledge and resources within these tools [48]. Paper‐based educational tools remain a valuable complement, particularly for birthing people with limited internet access, digital device availability, or lower levels of technological literacy. Developing and providing educational tools in a variety of languages is also important to meet the needs of populations of birthing people with limited English proficiency who may be at a higher health risk after a complicated pregnancy [49, 50]. However, the majority of educational tools in this scoping review were available only in English (*n = *7), indicating the need to develop and test postpartum educational tools for birthing people with limited English proficiency.

Finally, the outcomes of testing the educational tools in birthing people were limited to basic implementation, knowledge, emotional, and clinical outcomes. Further research should focus on comprehensively testing educational tools for birthing people after a complicated pregnancy for important outcomes related to implementation (e.g., patient satisfaction, patient motivation) and behavior outcomes (e.g., blood pressure control, healthy lifestyle choices) to better understand the efficacy of these educational tools for behavioral change.

### 4.1 Strengths and limitations

This scoping review included a robust database search strategy developed with an expert librarian and data assessment by two independent reviewers in accordance with the PRISMA‐ScR guidelines. The review provides a current evidence base and analysis of publications on patient education after a complicated pregnancy and accompanying educational tools. Our team contextualized the findings within a comprehensive theory of behavior change using the COM‐B framework to examine barriers and facilitators affecting the engagement of postpartum birthing people in their postpartum care and recovery.

Some patient educational tools were considered proprietary information by their authors and, therefore, were not available for analysis. Further compounding this limitation was that some authors did not respond to requests to share the educational tools for analysis or no longer had access to the educational tools. Therefore, COM‐B analysis was limited to educational tools available through the publications or responding authors.

The publications and educational tools included in this review were limited to those in English, Spanish, or French. Literature published outside of the United States was included; however, these findings may be more difficult to compare and analyze due to differences in healthcare settings, access to postpartum care and resources, and cultural contexts. This review identified several common themes around knowledge gaps, barriers, and facilitators of engagement in postpartum care and recovery, such as access to care, stigma, social support systems, and lack of medical awareness. These findings may have some generalizability despite the different cultural and health settings of the included publications and educational tools.

### 4.2 Next steps

This scoping review serves as the theoretical foundation for the development of an educational tool for birthing people with HDPs. The identified gaps in the literature and postpartum educational tools provide an opportunity for an educational tool to be publicly accessible for birthing people, available in multiple languages and at an appropriate reading level, be multimodal (e.g., QR codes of short videos and brief handouts) and interactive, and address behavior change within a framework such as COM‐B, including a focus on self‐care and patient empowerment to overcome barriers to and enhance facilitators of postpartum care access. Such a tool should then be tested to ensure both efficacy and effectiveness.

In general, rigorous development and testing of educational tools for diverse populations of birthing people to address and prevent pregnancy‐related complications is needed, but has not yet been performed. Once developed and tested, educational tools should be made publicly available if they are to be useful. Our research team was only able to access approximately one‐third of the tools identified in the database search. Most of which required time and resources to gain access, which is not feasible for many birthing people or their healthcare providers.

Recent work from our group [51] represents an example of the type of tool that can be developed to address the gaps identified in this scoping review.

## 5 CONCLUSION

This scoping review demonstrates the paucity of tested, easily accessible postpartum educational tools that focus on pregnancy‐related complications and promote healthy behavior changes for postpartum care and recovery. Specifically, there are few tools that address adverse pregnancy outcomes, are accessible in multiple languages, and aim to change behavioral outcomes in postpartum patients. Structuring the design and implementation of future educational tools within a recognized framework for behavioral change may be important for their efficacy in strengthening engagement in postpartum care and recovery, as well as access to socioeconomic resources during and beyond the postpartum period. As most of the educational tools identified in this review were in English, our findings support the need to develop culturally and linguistically inclusive educational tools to support diverse populations of birthing people, including those most at risk of adverse pregnancy outcomes from historically marginalized communities.

## CONFLICT OF INTEREST STATEMENT

The authors declare no conflicts of interest.

## Supporting information



Supporting Information

## Data Availability

The data that support the findings of this study are available from the corresponding author upon reasonable request.
