# Supplementary material for: Educational tools that address pregnancy‐related complications in the postpartum period: A scoping review
Source: Pregnancy (Hoboken). 2026 Aug 3;2(5):e70327. doi: 10.1002/pmf2.70327 (PMC13602980; doi:10.1002/pmf2.70327)
Supplement: Supplementary file 1 [file PMF2-2-e70327-s001.docx]

*Appendix 1.* Search Strategy.

*PubMed*

(("Hypertension, Pregnancy-Induced"[Mesh] OR "Depression, Postpartum"[Mesh] OR "Pregnancy Complications"[Mesh:NoExp] OR "Stillbirth"[Mesh] OR "Diabetes, Gestational"[Mesh] OR "Obstetric Labor Complications"[Mesh] OR "Placenta Diseases"[Mesh] OR "Pregnancy Complications, Cardiovascular"[Mesh] OR "Pregnancy Complications, Hematologic"[Mesh] OR "Pregnancy Complications, Neoplastic"[Mesh] OR "Pregnancy in Diabetics"[Mesh] OR "Pregnancy, Ectopic"[Mesh] OR "Puerperal Disorders"[Mesh] OR "Pregnancy, Prolonged"[Mesh] OR "Fetal Growth Retardation"[Mesh] OR (("Trauma and Stressor Related Disorders"[Mesh:NoExp] OR "Adjustment Disorders"[Mesh] OR "Stress Disorders, Traumatic"[Mesh:NoExp] OR "Domestic Violence"[Mesh] OR "Substance-Related Disorders"[Mesh] OR "Stress Disorders, Post-Traumatic"[Mesh] OR "Stress Disorders, Traumatic, Acute"[Mesh]) AND ("Pregnancy"[Mesh] OR "Peripartum Period"[Mesh] OR "Pregnant Women"[Mesh] OR "Pregnancy Trimesters"[Mesh] OR "Postpartum Period"[Mesh]))) OR (((preterm[tiab] OR premature[tiab] OR "pre term"[tiab] OR "pre mature"[tiab] OR difficult*[tiab] OR complicat*[tiab]) AND (birth*[tiab] OR lab?or[tiab] OR delivery[tiab])) OR preeclampsia[tiab] OR eclampsia[tiab] OR HELLP[tiab] OR "gestational diabetes"[tiab] OR "postpartum depression"[tiab] OR "postpartum anxiety"[tiab] OR "postpartum psychos*"[tiab] OR "post partum depression"[tiab] OR "post partum anxiety"[tiab] OR "post partum psychos*"[tiab] OR "fetal growth restrict*"[tiab] OR "fetal growth retard*"[tiab] OR stillbirth*[tiab] OR stillborn*[tiab] OR "still birth*"[tiab] OR "still born*"[tiab] OR ((hypertens*[tiab] OR HTN[tiab] OR diabet*[tiab] OR ectopic[tiab]) AND (pregnan*[tiab] OR gestation*[tiab])) OR "pregnancy complication"[tiab:~3] OR "complicated pregnancy"[tiab:~3] OR (("drug use"[tiab:~3] OR "substance use"[tiab:~3] OR "adjustment disorder*"[tiab] OR C-PTSD[tiab] OR CPTSD[tiab] OR PTSD[tiab] OR "trauma disorder"[tiab:~3] OR "trauma disorders"[tiab:~3] OR "post-traumatic disorder"[tiab:~3] OR "post-traumatic disorders"[tiab:~3] OR "posttraumatic disorder"[tiab:~3] OR "posttraumatic disorders"[tiab:~3] OR "domestic violence"[tiab] OR "intimate partner violence"[tiab] OR "spousal violence"[tiab] OR "spousal abuse"[tiab]) AND (pregnan*[tiab] OR gestation*[tiab] OR postpartum[tiab] OR puerperal[tiab] OR peripartum[tiab] OR "post partum"[tiab] OR "expectant mother*"[tiab])))) AND ("Patient Education as Topic"[Mesh] OR "education"[Mesh Subheading] OR "Patient Education Handout" [Publication Type] OR "patient education"[tiab:~3] OR "educational program*"[tiab] OR "educational material*"[tiab] OR "patient guidance"[tiab:~3] OR Educat*[Title]) AND ("Postpartum period"[Mesh] OR "Depression, Postpartum"[mesh] OR "Puerperal Disorders"[Mesh] OR "Postnatal Care"[Mesh] OR postpartum[tiab] OR "post partum"[tiab] OR puerper*[tiab] OR postnatal[tiab] OR "Post natal"[tiab])

*Embase*

(('pregnancy disorder'/mj OR 'ectopic pregnancy'/exp OR 'labor complication'/exp/mj OR 'obstetric emergency'/de OR 'placenta disorder'/exp OR 'puerperal disorder'/exp OR 'intrauterine growth retardation'/exp OR ('substance use'/exp/mj OR 'drug dependence'/exp/mj OR 'domestic violence'/de OR 'partner violence'/exp OR 'battered woman'/exp) AND ('pregnancy'/exp OR 'pregnant woman'/exp OR 'puerperium'/exp OR 'postnatal care'/de)) OR (((preterm OR premature OR 'pre term' OR 'pre mature' OR difficult* OR complicat*) NEAR/3 (birth OR lab?or OR delivery)) OR preeclampsia OR eclampsia OR HELLP OR ((diabet* OR complicat* OR hypertens* OR HTN OR ectopic) NEAR/3 (pregnan* OR gestat*)) OR (((disorder* NEAR/3 (trauma* OR postrauma* OR adjustment)) OR PTSD OR CPTSD OR psychos* OR depress* OR anxiety OR 'mental health' ) AND (pregnan* OR postpartum OR 'post partum' OR 'post natal' OR postnatal OR puerper*)) OR ((domestic OR spous* OR partner) NEAR/3 (violence OR abuse)) OR 'still born' OR 'still birth' OR stillborn* OR stillbirth* OR ('fetal growth' NEAR/3 (retard* OR restrict*))):ti,ab,kw) AND ('postnatal care'/de OR 'puerperium'/exp/mj OR 'puerperal disorder'/exp OR (Postpartum OR postnatal OR peuper* OR ((post OR after) NEAR/2 (delivery OR lab?or OR partum OR natal))):ti,ab,kw) AND ('patient education'/exp OR 'psychoeducation'/exp OR 'self care education'/exp OR ((educat* OR inform*):ti OR (patient* NEAR/3 (educat* OR inform* OR resource*)):ti,ab,kw)) AND [embase]/lim

*CINAHL*

( MH ("Pregnancy Complications" OR "Childbirth, Premature" OR "Fetal Diseases+" OR "Labor Complications+" OR "Placenta Diseases+" OR "Pregnancy Complications, Cardiovascular+" OR "Pregnancy Complications, Hematologic" OR "Pregnancy Complications, Infectious+" OR "Pregnancy Complications, Neoplastic+" OR "Pregnancy Complications, Psychiatric+" OR "Pregnancy, Ectopic" OR "Pregnancy in Diabetes+" OR "Puerperal Disorders+" OR "Pregnancy, Prolonged" OR "Fetal Growth Retardation" OR (( "Domestic Violence+" OR "Substance Use Disorders+" OR "Stress Disorders, Post-Traumatic+") AND ("Pregnancy+" OR "Pregnancy Trimesters+" OR "Postnatal Period+"))) OR TI (((preterm OR premature OR "pre term" OR "pre mature" OR difficult* OR complicat*) N3 (birth OR lab?or OR delivery)) OR preeclampsia OR eclampsia OR HELLP OR ((diabet* OR complicat* OR hypertens* OR HTN OR ectopic) N3 (pregnan* OR gestat*)) OR (((disorder* N3 (trauma* OR postrauma* OR adjustment)) OR PTSD OR CPTSD OR psychos* OR depress* OR anxiety OR "mental health" ) AND (pregnan* OR postpartum OR "post partum" OR "post natal" OR postnatal OR puerper*)) OR ((domestic OR spous* OR partner) N3 (violence OR abuse)) OR "still born" OR "still birth" OR stillborn* OR stillbirth* OR ("fetal growth" N3 (retard* OR restrict*))) OR AB (((preterm OR premature OR "pre term" OR "pre mature" OR difficult* OR complicat*) N3 (birth OR lab?or OR delivery)) OR preeclampsia OR eclampsia OR HELLP OR ((diabet* OR complicat* OR hypertens* OR HTN OR ectopic) N3 (pregnan* OR gestat*)) OR (((disorder* N3 (trauma* OR postrauma* OR adjustment)) OR PTSD OR CPTSD OR psychos* OR depress* OR anxiety OR "mental health" ) AND (pregnan* OR postpartum OR "post partum" OR "post natal" OR postnatal OR puerper*)) OR ((domestic OR spous* OR partner) N3 (violence OR abuse)) OR "still born" OR "still birth" OR stillborn* OR stillbirth* OR ("fetal growth" N3 (retard* OR restrict*))) ) AND ( (MH ("Depression, Postpartum" OR "Postpartum Psychosis" OR "Postpartum Hemorrhage" OR "Alopecia, Postpartum" OR "Postnatal Period+" OR "Postnatal Care+") OR TI (Postpartum OR postnatal OR peuperal OR ((post OR after) N2 (delivery OR lab?or OR partum OR natal))) OR AB Postpartum OR postnatal OR peuperal OR ((post OR after) N2 (delivery OR lab?or OR partum OR natal))) ) AND ( (MH ("Health Education" OR "Patient Education" OR "Patient Discharge Education" OR "Information Resources" OR "Pamphlets" OR "Pregnancy Complications+/ED") OR TI (educat* OR resource* OR inform*) OR AB (patient N3 (educat* OR inform* OR resource*))) )
